# Changes in lower limb muscle strength and corresponding gait changes following hip fractures in older adults: a narrative review

**DOI:** 10.3389/fmed.2026.1880514

**Published:** 2026-08-05

**Authors:** Wei Wang, Yang Liu, Ce Wang, Yaomin Li, Weiguo Xu

**Affiliations:** 1 Department of Rehabilitation, Tianjin University Tianjin Hospital, Tianjin, China; 2 Tianjin Hospital, Tianjin University, Tianjin, China; 3 Department of Geriatrics, Tianjin Medical University General Hospital, Tianjin, China

**Keywords:** gait, hip fracture, muscle strength, older adults, rehabilitation

## Abstract

This narrative review synthesizes available evidence on changes in lower limb muscle strength and corresponding gait alterations following hip fracture in older adults, aiming to provide a biomechanically grounded framework for precision rehabilitation. A structured literature search was conducted across major electronic databases (PubMed, Scopus, Web of Science, Embase, and the Cochrane Library) from inception to January 31, 2026, and findings were synthesized descriptively. The evidence indicates that muscle strength decline exhibits group-specific patterns: hip abductor weakness is associated with pelvic instability and Trendelenburg gait; quadriceps atrophy correlates with knee instability and limited flexion-extension; and weakness of the gluteus maximus and calf muscles appears linked to reduced stride length and diminished propulsive force. Recovery of muscle strength and gait typically follows a trajectory of rapid decline, gradual improvement within the first six months, and long-term persistence of sub-baseline levels, with bilateral asymmetry often lasting 1–2 years post-surgery; gait speed, stride length, and symmetry frequently remain impaired despite rehabilitation. These findings suggest that biomechanically informed, muscle group-specific training—guided by individual gait analysis—may enhance functional recovery and reduce fall risk, encouraging clinicians to move beyond generic lower-limb strengthening programs toward targeted, mechanism-based interventions.

## 1 Introduction

Hip fractures in older adults (≥65 years) are characterized by high incidence, high mortality, high disability rates, and high costs, imposing a heavy burden on patients themselves, their families, public health, and socioeconomic systems (1–3). With the aging global population, the incidence of this disease continues to rise (4, 5). Research findings indicate that performing hip fracture surgery within 24 to 48 h after admission reduces mortality rates, decreases complications, and shortens hospital stays (6, 7). However, patients with fractures experience loss of mobility after surgery, which can lead to complications such as muscle atrophy, severe osteoporosis, and decubitus pneumonia, significantly reducing their quality of life. The mortality rate among older patients within the first year after fracture reaches as high as 30%.

Among survivors, fewer than half regain pre-injury functional capacity (8, 9). Furthermore, 20% of older individuals who lived independently at home prior to a hip fracture are unable to live independently afterward (10–12).

Ninety percent of hip fractures in older adults are caused by simple falls, with most falls occurring while walking (13, 14). Older patients with hip fractures experience a higher recurrence rate and increased incidence of injurious falls due to altered lower limb muscle strength and gait patterns. Decreased muscle strength around the hip joint is often associated with limping or asymmetrical gait, which may contribute to secondary damage to other lower limb joints. The knee extensor and flexor muscles are the core muscle groups responsible for maintaining knee stability. Weakness in these muscles is associated with an increased risk of recurrent falls and subsequent hip fractures in older adults. Targeted postoperative rehabilitation exercises can effectively restore lower limb strength, reduce the risk of falls, and decrease the likelihood of recurrent fractures (15, 16). Given the considerable heterogeneity in assessment tools, patient populations, and follow-up durations across published studies, we adopted a narrative review approach to integrate and critically appraise the available evidence. This report provides a broad narrative synthesis and critical discussion of the existing research literature on the dynamic evolution of lower limb muscle strength and gait following hip fractures in older adults. By systematically categorizing changes across specific muscle groups—including hip abductors, adductors, extensors, flexors, quadriceps, hamstrings, and calf muscles—and linking these deficits to corresponding gait deviations, this review aims to offer clinicians, rehabilitation therapists, and researchers a structured conceptual framework for understanding the biomechanical basis of functional recovery and for designing targeted, muscle-group-specific interventions following hip fractures in older adults.

## 2 Literature search strategy

Given the narrative nature of this review, we did not aim to conduct a systematic review or adhere to PRISMA guidelines. Nevertheless, to ensure comprehensive coverage of relevant literature, we performed a structured literature search in the following electronic databases in February 2026: PubMed (MEDLINE), Scopus, Web of Science, Embase (Ovid), and the Cochrane Library. The search period covered from inception of each database to January 31, 2026, and was restricted to English-language publications.

The search strategy combined three core conceptual domains using the Boolean operator “AND”: (1) hip fracture (e.g., “hip fracture,” “proximal femoral fracture,” “femoral neck fracture”); (2) muscle strength (e.g., “muscle strength,” “sarcopenia,” “quadriceps strength,” “hip abductor strength”); and (3) gait and mobility (e.g., “gait,” “gait speed,” “stride length,” “balance,” “mobility”). Within each domain, synonyms and related terms were connected with “OR”. Exclusions: pathological/periprosthetic/high-energy fractures; age < 65 years or no subgroup data; case reports/series (< 10), editorials, conference abstracts, or protocols; low-quality articles (insufficient data or full-text unavailable). In addition, we manually screened the reference lists of all included studies and relevant review articles to identify potentially eligible publications not captured by the database searches.

## 3 Changes in lower limb muscle strength following hip fractures in older adults

### 3.1 Hip fractures and sarcopenia

Muscle atrophy is particularly common among older patients, especially following hip fractures, which may impair muscle regeneration and hinder functional recovery. Prolonged immobilization required after hip fractures restricts movement, causing muscles to remain inactive for extended periods and thereby exacerbating muscle wasting (17, 18). When talking about functional changes after hip fractures, sarcopenia is a fundamental concept that cannot be ignored.

**Figure 1:**
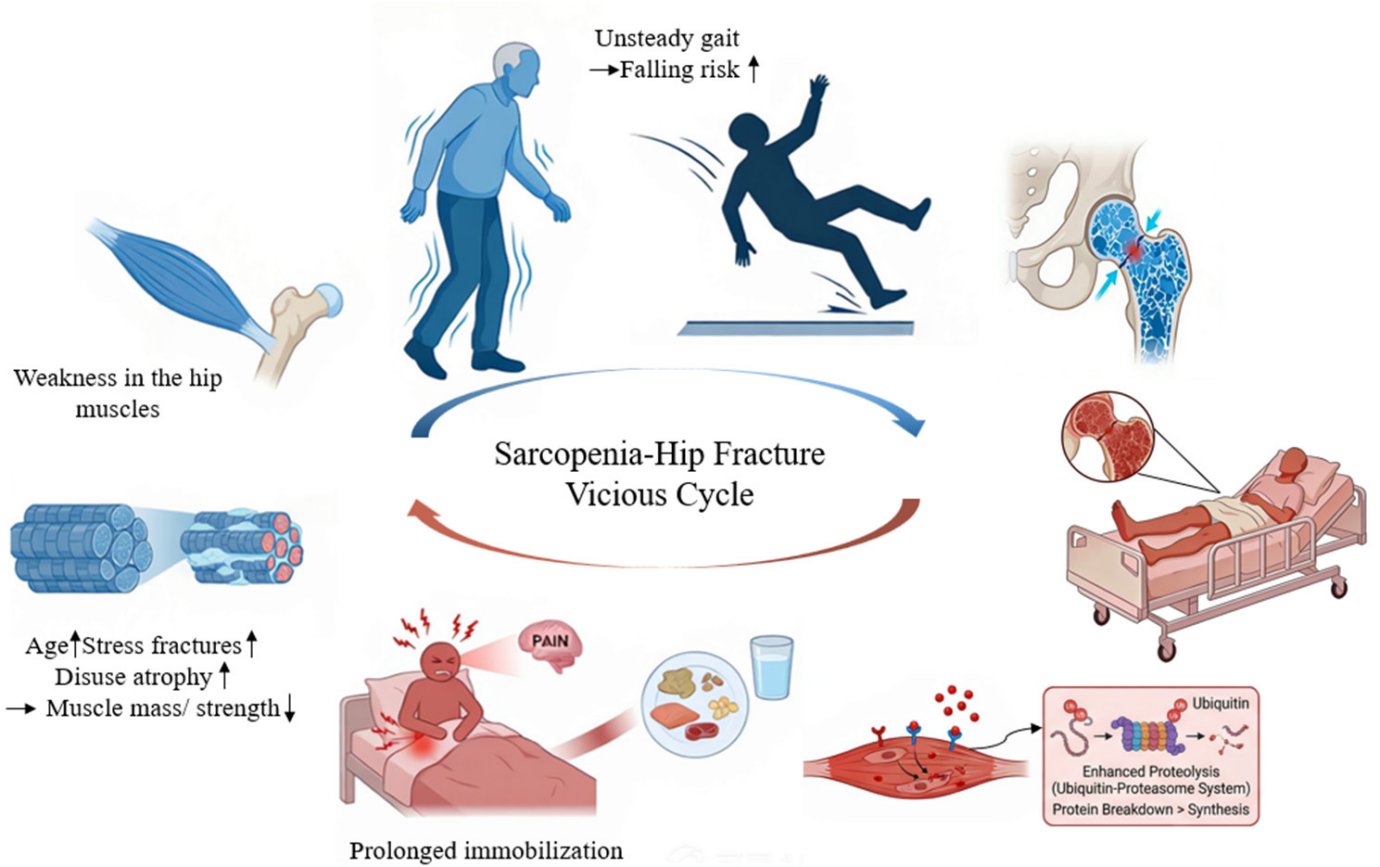
Diagram of the Vicious Cycle Between Sarcopenia and Hip Fractures.

Sarcopenia is an age-related syndrome characterized by progressive, widespread reduction in skeletal muscle mass, decreased muscle strength, and/or diminished physical function. In the older adult population, sarcopenia itself is a key factor contributing to increased risks of falls and osteoporotic fractures. Muscles are not only the organs responsible for movement but also vital tissues that maintain postural stability, absorb impact, and protect bones. When muscle mass declines, the body's ability to maintain balance and respond quickly to unexpected jolts diminishes, which may be associated with an increased risk of falls. Following a hip fracture, muscle loss is further accelerated, creating a vicious cycle (19, 20). The fracture injury itself, surgical stress, prolonged postoperative bed rest, activity limitations due to pain, and the exacerbation of catabolic states collectively lead to rapid muscle atrophy and strength decline in the lower limbs, particularly in the affected side. Multiple prospective studies indicate that older adults with poor muscle condition (low muscle mass, size, strength, or density) exhibit impaired balance, gait disturbances, and a higher risk of falls and fractures (21–24). Patients with lower limb muscle weakness and greater disability in daily living activities have been found to be at higher risk of falls, according to Lloyd et al. (25) (See Figure 1).

Lower limb muscle strength is a significant predictor of long-term survival in older patients with hip fractures. Kristensen et al. (26) demonstrated that patients with lower maximum isometric quadriceps strength on the non-fractured side during the acute postoperative period (at discharge) exhibited significantly higher 1-year mortality rates compared to those with higher strength (21.7% vs. 2.5%, *P* < 0.001), with a hazard ratio of 9.8 (95% CI = 2.2–43.0, *P* = 0.002). Therefore, rehabilitation following a hip fracture is largely a battle against muscle loss, rebuilding muscle function, and breaking this vicious cycle. Understanding the specific patterns of lower limb muscle strength changes after a fracture is crucial for developing effective rehabilitation strategies (See Table 1).

**Table 1:** Changes in lower limb muscle strength following a hip fracture.

Study	Study design	Study population (*n*, sex)	Mean age (years)	Assessment method	Muscle groups	Follow-up period	Operated side result	Healthy side result	Operated/Healthy (%)
Stasi et al. (44)	Cross-sectional	96 (24 M, 72 F)	77.53 ± 4.24	HHD	HAB	3 months	73.44 ± 15.57 N	Not reported	78.27 ± 2.06%
Ivanova et al. (35)	Prospective cohort	9 (0 M, 9 F)	71.4 ± 3.9	HHD	HAB, HAD, KE	1 week			HAB: 44.4% HAD: 61.2% KE: 49.3%
						6 months			HAB: 70.1% HAD: 80.6% KE: 63.2%
Chen et al. (38)	Prospective observational	30 (11 M, 19 F)	82.43 ± 7.35	DXA	LLM	1 year	Mean LLM loss: 5.85 ± 11.00 %		
Jeon et al. (75)	Cross-sectional	59 (16 M, 43 F)	79.2 ± 9.1	ARM	HAB, QUAD, KF	6 weeks	QUAD: 27.9 ± 12.6 Nm KF: 18.3 ± 8.5 Nm	QUAD: 44.4 ± 15.5 Nm KF: 22.6 ± 9.8 Nm	QUAD: 62.8% KF: 81.0%
Umehara et al. (17)	Cross-sectional	90 (22 M, 68 F)	80.1 ± 6.9	HHD	QUAD	6 months	1.13 ± 0.53 Nm/kg	1.87 ± 0.42 Nm/kg	60.4%
Kronborg et al. (32)	Prospective cohort	36 (9 M, 27 F)	79.4 ± 8.3	HHD	QUAD	Early post-op to discharge			Early post-op: 50 ± 33.6% Discharge: 68 ± 25.2%
Kristensen et al. (26)	Prospective observational	150 (36 M, 114 F)	78.7 ± 7.8	HHD, CSGD	QUAD	1 week (mean 8.2 days)	0.57 ± 0.32 Nm/kg	1.04 ± 0.41 Nm/kg	54.8%
Sajiki-Ito et al. (16)	Retrospective study	17 (4 M, 13 F)	84 (median)	HHD	QUAD	1 week	5.5 kgf [IQR, 2.0–8.0]	10.7 kgf [IQR, 6.1–24.0]	51.4%

HHD, handheld dynamometer; DXA, dual-energy X-ray absorptiometry; ARM, air-resistance weight machine; CSGD, custom-made strain-gauge dynamometer; HAB, hip abductors; HAD, hip adductors; KE, knee extensors; KF, knee flexors; QUAD, quadriceps; LLM, lower limb muscles; HAS, hip strength asymmetry; HHD, hand-held dynamometry; DXA, dual-energy X-ray absorptiometry; HSA, hip strength asymmetry.

### 3.2 Combined effects of systemic muscle decline and localized muscle atrophy

Research indicates that after age 30, muscle mass decreases by approximately 3–8% every decade, with the rate of decline accelerating even further after age 60 (27–30). Patients with hip fractures in older adults commonly exhibit insufficient muscle functional reserves prior to fracture. Multiple studies have confirmed that the prevalence of sarcopenia is extremely high among this patient population. Following surgery, patients enter an acute catabolic phase. Combined with factors such as bed rest, immobilization, pain, and inadequate nutritional intake, this leads to rapid muscle loss. This muscle loss occurs on two levels: Systemic muscle atrophy, where muscle mass and strength decline even in non-injured limbs or upper extremities due to systemic inflammatory responses and catabolic states; Localized disuse atrophy, where the affected lower limb experiences more severe muscle wasting due to immobilization and avoidance of weight-bearing. Disuse atrophy appears to be a major contributor to the significant imbalance in muscle strength between the affected and unaffected sides.

### 3.3 Long-term patterns of muscle strength changes following hip fractures

Following hip fractures, lower limb muscle strength follows a typical trajectory of rapid decline, gradual recovery, and long-term sub-baseline levels, exhibiting muscle group-specific variations and bilateral asymmetry (31). The early postoperative period following fracture surgery is characterized by rapid muscle strength decline. Within one week of fracture, muscle strength in the affected lower limb may decrease to 50% of the unaffected side, and in some frail older patients, it may even fall below 40% (32, 33). During this phase, older patients often exhibit “self-imposed immobilization” due to poor pain tolerance and weakened balance, significantly reducing lower limb activity even without strict bed rest, leading to rapid disuse muscle atrophy. Research by D'Adamo et al. (34) revealed marked changes in body muscle mass within 3–10 days after the hip fracture. Between 10 days and 2 months post-fracture, total body weight decreased by 1.95 kg, with muscle mass declining by 1.73 kg. Ivanova et al. measured muscle strength in the hip adductors, hip abductors, and knee extensors using a Lafayette hand-held dynamometer. They found that 1 week after hip fracture surgery, the isometric strength of these muscles decreased by 38.8%, 55.6%, and 50.7%, respectively, compared to the unaffected side. 6 months after hip fracture, muscle strength in these muscles had significantly increased compared to 1 week post-surgery, yet remained markedly weaker than the unaffected side (35).

**Figure 2:**
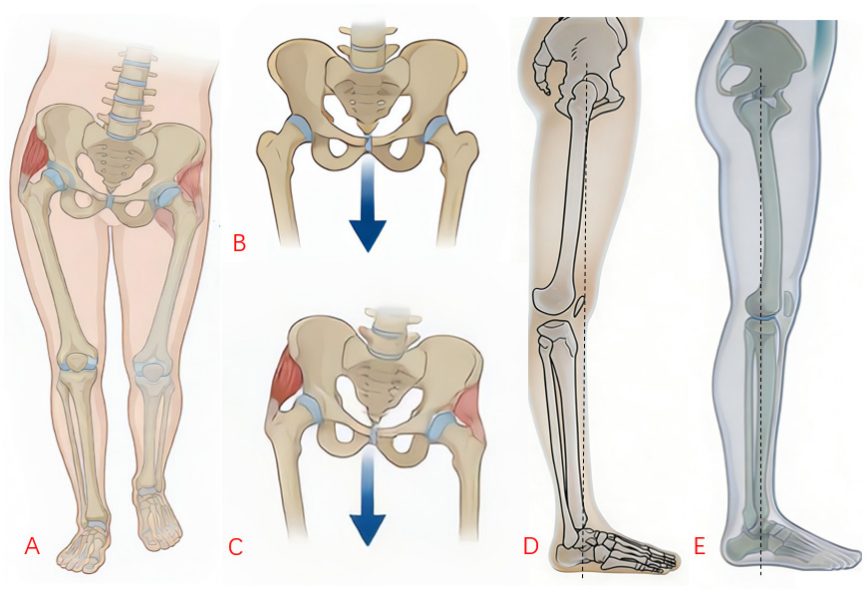
Muscle weakness and abnormal gait. **(A)**, Weakness of the hip abductors leading to a Trendelenburg gait; **(B)**, Normal pelvis, the blue arrow indicates the direction of gravity; **(C)**, Weakness in the hip abductors causes pelvic tilt; **(D)**, Weakness in the quadriceps muscles leads to hyperextension of the knee joint; E, Proper standing posture.

As pain subsides and rehabilitation training begins, muscle strength enters a gradual recovery phase, showing significant improvement within the first six months. Thereafter, the rate of improvement slows considerably, reaching a plateau (36). Before reaching the plateau phase, older patients require a longer period for muscle strength recovery, which can extend up to 12 months, and the overall muscle strength they can regain is generally inferior to that of younger patients (36). Muscle weakness in the lower limbs following hip fractures in older adults is persistent. Even 1 to 2 years after the fracture, muscle strength on the affected side may remain 10% to 20% lower than on the unaffected side, and this difference shows no significant correlation with the time elapsed since the fracture (37). The core hazard of long-term muscle weakness lies in disrupting the body's balance regulation capacity and gait stability, potentially contributing to a vicious cycle of muscle imbalance, fall, and refracture. Chen et al. assessed changes in body composition among hip fracture patients 1 year post-surgery. They found that only 43% of patients regained pre-fracture functional capacity within 1 year. While patients experienced increases in body mass index (BMI) and total body fat, they also suffered an average loss of 4.63% in skeletal muscle mass across the limbs. Notably, patients with sarcopenia exhibited a significantly higher loss compared to those without sarcopenia (9.18% vs. 1.15%) (38). Furthermore, since this study selected test-eligible individuals from a cohort of hip fracture patients, the actual muscle loss is likely higher than 4.63%. These studies demonstrate that treatment or rehabilitation training targeting muscle mass reduction following fracture surgery should be initiated as early as possible (See Figure 2).

### 3.4 Differential analysis of key muscle groups in the lower limb

#### 3.4.1 Hip abductor muscles

Normal hip joint movement relies on the constraints of ligaments and muscles. Research by Resende et al. (39) indicates that fatigue and weakness in the hip muscle groups can compromise the stability of the core muscle groups. The muscles surrounding the hip, particularly the hip abductors, play a crucial role in maintaining the proper position of the acetabulum and femoral head (40, 41). The hip abductor muscles primarily consist of the tensor fasciae lata, gluteus medius, and gluteus minimus. These muscles ensure pelvic stability during walking while also driving hip internal and external rotation as well as abduction (40, 42, 43). When this muscle is weak, during walking, as the unaffected leg lifts (swing phase), the gluteus medius on the affected side (support phase) cannot effectively stabilize the pelvis. This causes the pelvis to tilt toward the unaffected side, resulting in the characteristic “Trendelenburg gait” or “gluteus medius gait.” This gait pattern is not only inefficient, increasing the body's oxygen consumption, but also severely compromises balance, making postoperative patients prone to recurrent falls. Following hip fracture surgery, older patients exhibit significantly reduced strength in the hip abductor muscles on the fractured side compared to the unaffected side (31, 44, 45). Following a hip fracture, an asymmetric gait develops due to unequal muscle strength in both hip joints. This causes the peak coronal plane movement of the center of gravity during patient movement to exceed normal levels. If this peak coronal plane movement becomes excessively large, it becomes a risk factor for falls (46, 47). Chang et al. investigated the effects of hip abductor torque on kinematic and kinetic gait parameters, noting that insufficient hip abductor strength during walking leads to excessive pelvic drop on the swing side and a shift in the center of gravity toward the swing side. This results in increased load on the medial knee joint of the standing lower limb, raising the risk of medial tibiofemoral osteoarthritis in the standing lower limb. They further proposed that as hip abductor strength increases, the risk of medial tibiofemoral osteoarthritis decreases accordingly (48) (See Table 2).

**Table 2:** Changes in walking speed following a hip fracture.

Time point	Gait speed (m/s)	Characteristics of the stage
Pre-fracture	1.0–1.4	Reference values for healthy older adults (79, 83)
Postop. week 1	Not measurable	Severely impaired
Postop. 1–6 months	0.4–0.7	40–60% lower than healthy peers (15, 73)
Postop. 6–12 months	0.7–1.0	Plateau, minimal improvement (36, 76)
Postop. ≥12 months	0.7–1.2	10–20% lower than healthy peers (9, 38)

From a clinical rehabilitation perspective, the contralateral cane is an effective assistive device for compensating hip abductor insufficiency and alleviating the Trendelenburg sign. Its mechanism lies in the external moment generated by the cane's ground reaction force on the contralateral side, which counteracts pelvic tilting and thereby offloads the affected hip abductors. Neumann's (49) study demonstrated that in patients after hip surgery, contralateral cane use reduced electromyographic activity of the affected hip abductors by 31.1%, with a further reduction of 42.3% during forceful pushing. Similarly, Vargo et al. (50), in a single-leg stance experiment, confirmed that contralateral cane support lowered hip abductor activity to 66% of that during unsupported stance. Therefore, instructing patients in the proper use of a contralateral cane not only reduces muscle load and pain on the affected side, but also helps correct Trendelenburg gait and lowers fall risk, making it an important strategy in postoperative rehabilitation (51).

#### 3.4.2 Hip adductor muscles

The hip adductor muscles consist of the adductor magnus, adductor longus, adductor brevis, pectineus, and gracilis muscles, playing a crucial role in stabilizing and controlling the pelvis. Beyond providing adduction, the hip adductor group generates significant tension while stabilizing the hip joint and controlling linear movement of the lower limb (52). The hip adductors are key muscles controlling lateral balance. Weakened muscle strength is associated with reduced lateral stability, diminished force generation, and speed in protective lateral stepping, which may increase the risk of lateral falls (53). Additionally, when hip adductor muscle strength is insufficient, the likelihood of developing groin or hip pain increases. Older patients with hip fractures should focus on strengthening their hip adductor muscles to prevent potential future pain risks (54).

Recent studies have further advanced our understanding of adductor muscle dysfunction. Ling et al. found that hip adductor density was significantly lower in patients with hip fractures than in non-fracture controls, and this difference remained significant after adjusting for age, sex, and body mass index, suggesting that adductor weakness may be a pre-existing risk factor for fracture (55). Porto et al. indicated that adductor peak torque also significantly contributes to static single-leg standing balance in community-dwelling older adults (56). Morcelli et al. quantified age-related declines, showing that older women exhibited a 52% lower adductor peak torque and a 71% lower rate of torque development than younger women (57). These age-related deficits are further exacerbated after fracture by immobilization and disuse, leading to asymmetric adductor strength on the affected side, compromising frontal-plane stability during the mid-stance phase of gait, and increasing the risk of lateral falls and contralateral re-fracture. Therefore, rehabilitation training should concurrently address both maximal adductor strength and rapid force-generating capacity, while also focusing on improving neuromuscular activation efficiency.

#### 3.4.3 Gluteal muscles/hip extensors

During hip flexion, the force generated by the hip extensor muscles is frequently utilized to accelerate the body upward and forward, enabling athletes to perform actions such as sprinting, jumping, and rapid changes in direction. These muscles also play a significant role in cushioning impacts during landing maneuvers (58).

Following a hip fracture, weakened hip extensor muscle strength diminishes the ability to perform the aforementioned activities, potentially leading to knee joint injuries and lower back pain (59). The gluteus maximus, as the strongest muscle in the human body, serves as the primary engine for hip extension and propelling the body forward. When gluteus maximus strength diminishes, it directly results in weakness during the push-off phase of the gait cycle, which is associated with a significant reduction in stride length and a consequent decrease in gait speed.

Research by Horstmann et al. (60) indicates that hip muscle strength and endurance are significantly correlated with the severity of gait abnormalities. Furthermore, compared to hip abductors and adductors, hip extensors and flexors exert a more pronounced influence on gait. Inaba et al. (61) found that as the side step distance increased, the hip extension moment increased significantly, while the hip abduction moment showed no significant increase. Research by Shimokochi et al. (62) suggests that hip abduction function does not appear to be a key factor in lateral movement, whereas faster hip extension is crucial for more explosive movements in the coronal plane. These two studies demonstrate that when the body loses balance and falls sideways, sufficiently strong hip extensors can promptly protect the body through lateral movement to maintain equilibrium. However, following a hip fracture, this ability diminishes, resulting in a greater risk in such scenarios. The hip flexor muscles are a group of muscles located at the front of the hip joint. They are primarily responsible for hip flexion and maintaining pelvic stability. These muscles work together to perform actions such as walking, running, and lifting the legs, and collaborate with the core muscles to maintain body balance. Research by Akalan et al. (63) indicates that weakened hip flexor strength reduces average hip flexion speed by 21.5%, leading to decreased gait speed and a stiff-knee gait pattern. Therefore, treatment protocols that weaken hip flexors are not recommended for addressing hip flexion gait.

#### 3.4.4 Quadriceps

The muscles surrounding the knee joint are crucial structures for maintaining knee stability, particularly the quadriceps femoris. As the core muscles preventing kneeling falls, they cushion landing impact through eccentric contraction during the initial support phase and maintain stability by keeping the knee slightly bent during the mid-phase. When strength diminishes, the knee may hyperextend upon landing, a phenomenon often referred to as “buckling knees” phenomenon. This is especially prone to occur during uphill/downhill movements or turns, where loss of knee control can easily lead to falls. Additionally, insufficient quadriceps strength is a primary cause of diminished ability in the critical movement of rising from a seated position.

Following hip fractures, atrophy of the quadriceps femoris muscles occurs particularly rapidly and severely. Imaging studies using ultrasound and CT reveal significant reductions in muscle thickness and cross-sectional area, especially in the rectus femoris and vastus medialis and lateralis muscles, with notably pronounced declines in their mass parameters. As one of the primary muscle groups in the lower limbs, changes in quadriceps mass are closely associated with postoperative functional recovery. Research by Sparks et al. (64) indicates that quadriceps atrophy and functional decline not only delay the recovery process but may also lead to postoperative complications. During the recovery of lower limb joint function following hip fractures, the quadriceps femoris muscle group—including the rectus femoris, vastus lateralis, and vastus medialis—plays a crucial role. These muscle components work synergistically to provide dynamic stability and balance at the hip and knee joints, which is essential for normalizing postoperative gait and restoring function. A reduction in the thickness and cross-sectional area of the rectus femoris directly diminishes its knee extension capacity and stability, thereby limiting the patient's mobility and prolonging the rehabilitation period (65). Reduced thickness of the vastus lateralis and vastus medialis muscles may also contribute to an imbalance in hip abduction and adduction function. During walking, this imbalance has been associated withgait instability, which may elevate the risk of falls and subsequent re-injury (66, 67). Quadriceps strength directly impacts knee joint stability. Incorporating quadriceps functional training into rehabilitation programs for hip fracture patients enhances knee stability and effectively reduces varus stress, thereby promoting joint function recovery (15, 37, 68).

#### 3.4.5 Hamstrings

The hamstrings serve as the primary antagonists to the quadriceps, while also functioning as important hip extensors and knee flexors. Few studies have specifically examined the relationship between hamstring function and gait speed; however, it is undeniable that the hamstrings play an equally vital role in maintaining gait stability. Stability and coordinated movement of the knee joint during walking require the synergistic action of surrounding muscles, particularly the balanced coordination between the quadriceps and hamstrings. For instance, when the heel first contacts the ground, the quadriceps contract to prevent excessive knee flexion, while the hamstrings undergo eccentric contraction to prevent knee hyperextension and anterior leg displacement. When hamstring strength diminishes, excessive anterior swing of the lower leg during the swing phase occurs, and the knee joint fails to effectively resist flexion during the stance phase. This has been associated with impaired gait coordination and increased risk of tripping (69). Lloyd et al. (70) utilized an electromyography-driven neuromuscular biomechanical model to determine that knee joint stability is highest when the quadriceps and hamstrings generate knee extension and flexion moments, respectively, followed by when the quadriceps and hamstrings co-contract. These studies collectively demonstrate the hamstrings' crucial role in maintaining knee joint stability. Simultaneously, as synergistic muscles for hip extension, hamstring weakness can also impair push-off ability during gait. Maintaining an appropriate hamstring-to-quadriceps strength ratio is essential for preventing sports injuries and preserving joint health—a principle equally applicable to the rehabilitation process of older hip fracture patients.

#### 3.4.6 Calf muscles

The calf muscle group, as a key component of the lower limb kinetic chain, performs core functions in foot-ankle stability, gait propulsion, and balance regulation. When hip fractures happen, the activation of these muscles is greatly lowered due to prolonged postoperative walking or abnormal gait patterns, such as immediate full-foot contact after heel strike with no normal heel-to-toe progression, making disuse atrophy inevitable. However, due to their anatomical distance from the fracture site and the ability to maintain partial muscle activity through minimal movement during bed rest, the degree of muscle loss is relatively mild.

The calf muscle group can serve as a predictor of walking ability following hip fracture surgery. A case-control study by Savio et al. (71) (46 cases) demonstrated that insufficient calf muscle strength (low calf circumference) is a significant risk factor for the inability to walk independently at 6 weeks postoperatively [OR (95% CI): 6.175 (1.589–23.993)]. The calf muscle group is responsible for plantar flexion of the ankle joint, serving as the primary joint muscle group generating forward propulsion during walking. The restoration of ankle plantar flexion torque is considered one of the key factors in increasing stride length and gait speed. When the triceps surae muscle is weak, the patient's “toe-off” at the end of the stance phase becomes very weak or even absent. This appears to contribute to insufficient propulsive force generated by the affected leg, which is considered one of the most direct biomechanical factors associated with reduced stride length and slowed gait speed post-surgery. Many patients adopt a compensatory gait pattern characterized by dragging the leg, which is a classic manifestation of functional deficits in the calf muscle groups (72).

## 4 Evolution and recovery of gait patterns following hip fracture

Gait is the fundamental mode of human locomotion, representing a complex process requiring highly coordinated design of the nervous, muscular, and skeletal systems. Hip fractures inflict devastating damage upon this intricate system, resulting in a series of classic gait abnormalities. The degree of gait recovery serves as the most direct and crucial indicator for assessing the success of a patient's rehabilitation.

### 4.1 General deterioration in gait parameters

When attempting to stand and walk for the first time after surgery, patients typically exhibit a protective, unstable gait pattern characterized by slowness, asymmetry, and lack of coordination.

#### 4.1.1 Gait speed

Gait speed is considered the “sixth vital sign” for assessing health in older adults. It serves as a comprehensive indicator reflecting cardiopulmonary function, muscular strength, balance ability, and the integrative capacity of the nervous system (73). Following hip fracture surgery, the patient's gait speed will decrease significantly (31). This slowing appears to reflect multiple factors acting together: pain, fear-induced protective withdrawal of the affected limb, severe muscle weakness, impaired balance, and adjustments in motor control strategies by the central nervous system.

Due to its ease of measurement (10-meter walk test) and strong correlation with multiple functional indicators—including fall risk, hospital stay duration, quality of life, and even morbidity—gait speed has become one of the most commonly used and critical metrics for assessing functional recovery in hip fracture patients (74). Research indicates that gait speed is significantly correlated with osteoporosis status, balance ability, and lower limb strength (particularly hip abductor and knee extensor muscle strength). In Jeon et al.‘s study, patients' average gait speed at 1 month post-surgery was only 40% of that observed in healthy older individuals. Even 1 year post-surgery, gait speed remained difficult to restore to preoperative levels (75, 76). Abellan et al. investigated the relationship between gait speed and adverse outcomes in older adults, proposing that gait speed serves as a simple, safe, and convenient predictor of adverse outcomes. Individuals with slowed gait speed exhibited more than double the risk of mortality compared to those without slowed gait speed. Furthermore, fall rates are typically higher among older adults who have slow gait speed (77).

#### 4.1.2 Stride length and cadence

Stride length is another important spatial parameter that reflects walking function (78, 79). Following a hip fracture, patients experience a significant reduction in stride length, particularly on the affected side due to diminished push-off force. This is primarily caused by weakness in the gluteus maximus and ankle plantar flexor muscle groups, which fail to generate sufficient propulsive force during the late stance phase (31, 80). Gausden et al. (81) analyzed the postoperative gait of 72 patients with hip fractures and found that these patients exhibited a significant decrease in step frequency, prolonged double-leg stance time, shortened stride length, prolonged single-leg stance time, and asymmetrical gait patterns. Concurrently, the unaffected leg's stride length may also shorten as it rapidly contacts the ground to minimize weight-bearing time on the injured leg.

Changes in step frequency are more complex. In some cases, patients may compensate for a shorter stride length by increasing their step frequency in an attempt to maintain a certain gait speed. However, in most cases, step frequency decreases as overall physical ability declines (82, 83).

### 4.2 Gait symmetry

Symmetry is a key characteristic of healthy gait. Following hip fracture, gait symmetry is severely disrupted, manifesting as asymmetry in both spatiotemporal and dynamic parameters (84). Regarding temporal parameters, the most typical manifestation is a significant reduction in stance time on the affected leg, accompanied by a corresponding increase in swing time. Patients consciously or unconsciously shift weight from the affected leg to the unaffected leg as quickly as possible to alleviate pain and instability (85). This results in substantially prolonged stance time on the unaffected leg, which assumes greater weight-bearing and stabilizing responsibilities. Double-support time also increases, representing a compensatory strategy adopted by the body to enhance stability. In terms of spatial parameters, asymmetric stride length is highly prevalent, typically manifested as the unaffected leg taking longer strides than the affected leg (73). Additionally, to increase the base of support and enhance stability, patients may also exhibit increased step width. Dynamically, gait analysis using force plates reveals significantly lower peak vertical ground reaction forces in the affected leg compared to the unaffected leg. This indicates pronounced “limping” during walking, reflecting an inability to fully load weight onto the affected side. Joint moments also exhibit severe asymmetry, with significantly reduced extension and abduction moments at the hip, knee, and ankle joints on the affected side (86). This directly reflects the functional deficit in the corresponding muscle groups.

To objectively quantify the degree of gait asymmetry, researchers have developed a range of assessment metrics, including the symmetry index, the symmetry angle (87). In addition, the Weighted Universal Symmetry Index (wUSI) has been proposed as a comprehensive method to quantify three-dimensional ground reaction force asymmetries (88).

Thingstad et al. (73) provided reference values based on an analysis of 249 patients at 4 months after hip fracture surgery: mean step-length asymmetry was 15% (range 0–163%), step-time asymmetry averaged 10% (range 0–46%), and single-support time asymmetry averaged 14% (range 0–76%). Interventions such as strength training for the affected limb, gait retraining, and appropriate selection of walking aids can all mitigate these asymmetries. Quantitative symmetry assessment thus offers an objective tool for setting individualized rehabilitation goals and for identifying patients at risk of persistent functional disability.

### 4.3 Balance ability

Hip fractures severely impair patients' balance abilities. Performance is significantly reduced in both static balance tests, such as the one-leg stand with eyes open/closed, and dynamic balance tests, including the functional reach test and the timed up and go test.

Balance ability is fundamental to stable gait. Studies have repeatedly confirmed that balance ability is closely related to gait parameters (75). Balance training is considered an important component for improving gait speed and overall functional recovery.

Objective assessment tools include the Berg Balance Scale (BBS), Timed Up and Go (TUG), one-leg stance time, and force plate-derived postural sway measures (center of pressure displacement and velocity). A meta-analysis further confirmed that balance training significantly improves overall physical function (SMD = 0.59) and gait speed (SMD = 0.63) (89). Moreover, knee extensor weakness, balance deficits, and restricted ankle dorsiflexion range of motion collectively influence gait variability, underscoring the multifactorial nature of the balance–gait relationship (90).

Collectively, these findings position balance training as a core rehabilitation component for enhancing gait speed and promoting overall functional recovery. Objective measures—including the BBS, TUG, and force-plate-derived postural sway indices—not only help identify patients at risk for persistent gait impairment but also serve as quantitative benchmarks for monitoring rehabilitation progress and guiding therapeutic interventions.

### 4.4 Long-term recovery trajectory of gait

The recovery of gait function is a lengthy and variable process, with individual recovery trajectories differing from person to person, though certain general patterns emerge. The first six months post-surgery represent the “golden period” for functional recovery. During this stage, as pain subsides, fractures heal, and rehabilitation training is implemented, patients experience significant improvements in gait parameters such as gait speed and step frequency (91). The majority of objective functional recovery is essentially completed within the first six months following surgery (36).

Six months after surgery, the pace of functional recovery typically slows down, entering a plateau phase. However, subjective functional improvements may persist up to 9 months post-surgery or even longer (36). Some studies indicate that even 1 year after surgery, gait parameters and muscle strength continue to show gradual recovery in some patients (91).

Despite rehabilitation efforts, a discouraging reality remains: a significant proportion of patients never regain their pre-fracture gait function. 1 year post-surgery, only 30–50% of patients achieve independent walking (92, 93). Many patients require lifelong dependence on assistive devices such as walkers. Even those who regain independent walking often exhibit significantly reduced gait speed, stride length, and gait symmetry compared to healthy older individuals of the same age. One to two years after surgery, 40–70% of patients still require assistance from others to complete daily activities (11). Studies indicate that even years post-surgery, disparities persist between the affected and unaffected sides in terms of muscle strength, joint torque, and gait symmetry (91, 94). This persistent asymmetry not only impairs walking efficiency but may also serve as a long-term risk factor for overuse and degenerative changes in the unaffected joints.

## 5 Role of assistive devices in compensating for gait alterations

Given that 82.2% of older adults with hip fracture are able to use a cane by 4 weeks postoperatively, elucidating the impact of these devices on long-term gait recovery carries significant clinical importance (95). The choice of walking aid significantly influences gait symmetry. Ono et al. compared spatiotemporal gait parameters in patients with unilateral hip fractures when using a single T-cane, double T-canes, and walking poles (84). They found that acceleration indices of step symmetry in the vertical and anteroposterior directions were significantly larger when walking with walking poles compared to a single T-cane, leading to the conclusion that walking with walking poles might promote a more symmetrical gait style than walking with a single T-cane.

The biomechanical basis for these differences lies in the contralateral cane mechanism. Holding the cane in the hand contralateral to the affected hip reduces the demands on the hip abductor muscles by creating an external moment that counteracts pelvic drop (51). Electromyographic studies have demonstrated that contralateral cane use reduces hip abductor muscle activity by approximately 40% compared to walking without a cane (49, 96). However, a single T-cane may introduce its own compensatory strategies: patients may develop an asymmetrical upper-body lean toward the cane, which, while unloading the affected hip, can perpetuate frontal plane asymmetry (84, 97). Double canes or walking poles, by providing bilateral support, may offer a more balanced unloading pattern and encourage more symmetrical weight distribution over the long term (84, 98). Therefore, while cane use is beneficial for reducing pain, improving stability, and facilitating early ambulation, rehabilitation professionals should carefully consider the type of walking aid prescribed. Walking poles or bilateral canes may be preferable for patients in whom gait symmetry is a primary rehabilitation goal, whereas a single contralateral cane may suffice for those with milder asymmetry or greater upper-extremity limitations.

## 6 Research limitations

First, muscle strength assessment systems remain inconsistent, with a current lack of standardized evaluation tools for older patients. Significant variations exist in the assessment methods employed across different studies (e.g., isometric contraction tests, functional muscle strength scoring), making it difficult to compare research findings across studies. Second, most studies remain limited to describing spatiotemporal parameters (e.g., gait rate, stride length), while research delving into changes in gait dynamics (e.g., joint moments, power) and kinematics (e.g., joint angles, coordination patterns) and their precise correlations with specific muscle group strength deficits remains relatively scarce. This limits our deep understanding of compensatory mechanisms for abnormal gait, thereby affecting the precision of rehabilitation strategies. Finally, most studies focus their follow-up periods on 3 months, 6 months, or 1 year post-surgery, leaving little knowledge about the evolution of muscle strength and gait patterns in patients over several years or longer. The long-term effects of hip fractures may persist throughout life, making understanding their long-term trajectory crucial for developing lifelong health management strategies.

## 7 Conclusion

Hip fractures in older adults represent a critical pathway to frailty and functional decline. The rapid decline in lower limb muscle strength following fracture appears to be a fundamental factor underlying severe gait impairment and the difficulty in achieving full recovery. In terms of muscle strength, older patients with hip fractures exhibit a general decline in lower limb strength on the affected side compared to healthy individuals or the unaffected side. This decline is most pronounced in the quadriceps femoris and hip abductor muscle groups on the affected side. Post-hip fracture surgery, lower limb muscle strength follows a typical trajectory: rapid decline, gradual recovery, and long-term maintenance below baseline levels. This process also exhibits muscle group-specific variations and bilateral asymmetry. In terms of gait, patients commonly exhibit slowed gait speed, shortened stride length, severe gait asymmetry, and impaired balance. These issues often persist long-term, significantly impacting the quality of life for older patients. Almost every gait abnormality can be traced back to dysfunction in a specific muscle group or set of muscles. Therefore, rehabilitation therapy should not be a generic “lower-body strength training” program, but rather a targeted “precision approach” based on gait analysis. To operationalize this precision approach, the following core variables should be systematically assessed: (1) isometric strength of key muscle groups using handheld dynamometry, with emphasis on side-to-side asymmetry; (2) spatiotemporal gait parameters; (3) dynamic balance and mobility; and (4) weight-bearing symmetry, assessed via force plates or pressure-sensing walkways. Based on these assessments, targeted interventions can be prescribed: abduction strengthening with contralateral cane training for Trendelenburg gait; gluteus maximus and calf muscle training for shortened stride length; bilateral walking aids (walking poles or double canes) for pronounced temporal asymmetry; and task-specific balance training for impaired dynamic balance. This biomechanically grounded, assessment-driven approach is key to enhancing the efficiency and effectiveness of recovery in older patients following hip fracture.
